# Efficacy and safety of laser therapy for the treatment of retinopathy of prematurity: A protocol for systematic review of randomized controlled trials

**DOI:** 10.1097/MD.0000000000023282

**Published:** 2020-12-11

**Authors:** Fei Wang, Li-xia Hao

**Affiliations:** a Department of Ophthalmology; b Department of Neonatology, Yan’an University Affifiliated Hospital, Yan’an, Shaanxi, China.

**Keywords:** efficacy, laser therapy, retinopathy of prematurity, safety

## Abstract

**Background:**

Retinopathy of prematurity (ROP) is a potentially blinding eye disorder that primarily affects premature infants. Although a variety of managements are available for ROP, the efficacy is still unsatisfied. Studies have suggested that laser therapy (LT) may benefit ROP. However, no systematic review has addressed this topic. Thus, this systematic review aims to evaluate the efficacy and safety of LT for ROP.

**Methods:**

A comprehensive literature search will be performed from electronic databases (PubMed, EMBASE, AMED, Cochrane Library, WorldSciNet, Science online, Web of Science, Allied and Complementary Medicine Database, Chinese Biomedical Literature Database, and China National Knowledge Infrastructure) and other sources. The time is from the database construction to the present. Two investigators will independently carry out literature selection, data extraction and quality assessment. We will utilize RevMan 5.3 software for statistical analysis. Data synthesis will be conducted either as a narrative summary or meta-analysis. Statistical heterogeneity will be appraised using *I*^*2*^ test. If heterogeneity is low, pooled estimates will be calculated using a fixed-effects model. Otherwise, we will utilize a random-effects model to pool the data.

**Results:**

This study will provide up-to-date evidence on the efficacy and safety of LT for ROP, which may help to assess whether LT is effective and safe for ROP.

**Conclusion:**

This study will provide helpful evidence to determine whether or not LT is effective and safe for ROP, which may benefit both patients and clinicians.

**Study registration:**

osf.io/3tmnf.

## 1 Introduction

Retinopathy of prematurity (ROP) is a leading cause of visual impairment in premature infants that occurs because of the abnormal development of retinal vasculature at retina.^[1,2]^ It is estimated that about 28300 to 45600 infants with irreversible visual impairment due to ROP annually worldwide.^[3]^ Infants who weight 1250 g or less before 31 weeks gestation at delivery are more likely to develop ROP at highest risk.^[4]^

Although the pathogenesis of ROP is poorly understood, vascular endothelial growth factor (VEGF) is found to play an essential role in the ROP development,^[5,6]^ because dysregulation of VEGF causes abnormality in vasculogenesis and neovascularization.^[7–9]^ Over past few decades, treatment with laser therapy (LT) has been reported to manage such condition and prevent blindness by suppressing overproduction of VEGF in the retina.^[10–12]^ It also helps induce the regression of new vessels by ablating peripheral retina ischemic areas.^[11–13]^

Presently, numerous studies reported that LT can be utilized to treat ROP.^[14–28]^ However, all conclusions are drawn based on the individual clinical trial, and there are inconsistent conclusions.^[14–28]^ In addition, no systematic review specifically addresses this topic. Thus, the aim of this study is to systematically and comprehensively investigate the efficacy and safety of LT for ROP.

## 2 Methods and analysis

### 2.1 Study registration

We have registered this study on OSF (osf.io/3tmnf). It is being reported based on the Preferred Reporting Items for Systematic Reviews and Meta-Analysis Protocol statement guidelines.^[29]^

### 2.2 Eligibility criteria for study selection

Inclusion criteria will be created according to the description model of PICOS (participants, interventions, comparison, outcomes and study design). Inclusion criteria are:

1. Types of participants: preterm children with ROP (birth weights ≤1500 g and gestational age at birth <37 weeks)^[30]^ without limitation to the ethnic, regional, and sources.
2. Types of interventions: the experimental intervention must be LT alone. However, any treatments combined with LT will be limited.
3. Types of comparators: as for controls, patients who accepted any other interventions without restrictions will be considered for inclusion. However, we will not consider comparators involving any forms of LT.
4. Types of outcome measurements: the primary outcome is ROP persistence or recurrence. The secondary outcomes include ocular complications requiring retreatment, need for surgery, situation of the retinal periphery, quality of life (as measured by 36-Item Short Form Health Survey and any other scales), and complications.
5. Types of studies: all randomized controlled trials (RCTs) of LT in treating ROP without language limitation will be considered.

Exclusion criteria are as follows:

1. Case studies, quasi-RCTs, and non-RCTs will be excluded.
2. Besides retinopathy, participant also had other eye conditions.
3. Participants received other treatments that may affect treatment efficacy of LT.

### 2.3 Search strategy

The search strategy is developed by a specialist clinical librarian in expertise of systematic reviews. We will comprehensively search potential literature from electronic databases and literature sources.

#### 2.3.1 Electronic database sources

The literature will be searched in PubMed, EMBASE, AMED, Cochrane Library, WorldSciNet, Science online, Web of Science, Allied and Complementary Medicine Database, Chinese Biomedical Literature Database, and China National Knowledge Infrastructure. The search time of all databases is from the database construction to the present. The search terms include “retinopathy of prematurity”, “infant, premature”, “ROP”, “prematurity”, “retinopathy”, “retinopathies”, “retrolental”, “fibroplasias”, “fibroplasias”, “premature”, “extremely premature”, “preterm”, “extremely preterm”, “infant”, “infants”, “laser therapy”, “laser”, “lasers”, “low-level”, “high-level”, “therapy”, “treatment”, “intervention”, “management”, “randomized controlled trials”, “clinical trials as topic”, “random”, “randomly”, “control”, “comparator”, “allocation”, “placebo”, “blind”, “clinical study”, “clinical trials”, “controlled study”, and “controlled trial”. The complete Cochrane Library search strategy is presented in Table 1. We will adapt similar search strategies to other electronic databases.

**Table 1:** Search strategy for Cochrane Library.

Number	Search terms
1	MeSH descriptor: (retinopathy of prematurity) explode all trees
2	MeSH descriptor: (infant, premature) explode all trees
3	((ROP^∗^) or (prematurity^∗^) or (retinopathy^∗^) or (retinopathies^∗^) or (retrolental^∗^) or (fibroplasia^∗^) or (fibroplasias^∗^) or (premature^∗^) or (extremely premature^∗^) or (preterm^∗^) or (extremely preterm^∗^) or (infant^∗^) or (infants^∗^)):ti, ab, kw
4	Or 1–3
5	MeSH descriptor: (laser therapy) explode all trees
6	((laser^∗^) or (lasers^∗^) or (low-level^∗^) or (high-level^∗^) or (therapy^∗^) or (treatment^∗^) or (intervention^∗^) or (management^∗^)):ti, ab, kw
7	Or 5–6
8	MeSH descriptor: (randomized controlled trials) explode all trees
9	MeSH descriptor: (clinical trials as topic) explode all trees
10	((random^∗^) or (randomly^∗^) or (control^∗^) or (comparator^∗^) or (allocation^∗^) or (placebo^∗^) or (blind^∗^) or (clinical study^∗^) or (clinical trials^∗^) or (controlled study^∗^) or (controlled trial^∗^)):ti, ab, kw
11	Or 8–10
12	4 and 7 and 11

#### 2.3.2 Other literature sources

As for manual search, we will mainly retrieve for relevant literature from dissertations, ongoing trials, and conference abstracts.

### 2.4 Study identification

There are 2 investigators filtering out the literature records that clearly do not conform to the study. Any other disagreements regarding study identification between 2 investigators will be solved by a third investigator via discussion. All titles or abstracts of literature records will be screened and all unqualified documents will be excluded. After that, all remaining copies will be further strictly filtered out if they finally meet all eligibility criteria. Reasons for exclusion will be recorded at each stage. The process of study identification will be presented in the flowchart.

### 2.5 Data extraction

Two investigators will independently carry out data extraction using predefined standardized data extraction sheet. It consists of the following information of general information (such as title, first author, time of publication, etc.), study methods (study design, sample size, randomization, blinding, etc.), participant characteristics (race, gender, diagnostic criteria, eligibility criteria, etc.), intervention details, outcome measurements, safety, and important references. Any unclear information or missing data will be inquired by contacting primary authors. We will analyze available data using intention-to-treat analysis if those data can not be obtained.

### 2.6 Risk of bias assessment

Two investigators will independently utilize Cochrane risk of bias tool to assess study methodological quality for all included records.^[31]^ If there are different opinions, a third independent investigator will be invited to solve them through discussion.

### 2.7 Data synthesis

We will use RevMan 5.3 software for statistical analysis. Binary categorical outcome data will be calculated as risk ratio and 95% confidence intervals (CIs). Continuous outcome data will be expressed as mean difference or standardized mean difference and 95% CIs. The level of heterogeneity among included studies will be determined using *I*^*2*^ test.^[32]^ If value of *I*^*2*^ is 50% or less, low heterogeneity is considered, and a fixed-effect model is used.^[33]^ We will perform meta-analysis if sufficient eligible studies are included. Otherwise, if value of *I*^*2*^ is over 50%, high heterogeneity is considered, and a random-effect model is applied.^[34]^ We will carry out subgroup analysis to explore sources of heterogeneity. In addition, we will report outcome results as a narrative synthesis of study findings if there is still high heterogeneity after subgroup analysis. It will be conducted in accordance with the guidelines on the Conduct of Narrative Synthesis in Systematic Reviews.^[35]^

### 2.8 Strength of evidence

Two investigators will independently assess the strength of evidence for each outcome using Grading of Recommendations Assessment Development and Evaluation tool (GRADE).^[36]^ Any divergences will be resolved by a third investigator through consultation, and a final decision will be reached. We will summarize the results in a table based on the principle of GRADE.

### 2.9 Subgroup analysis

We will conduct subgroup analysis to identify any possible factors that are responsible for the high heterogeneity based on the different treatments, comparators, and outcomes.

### 2.10 Sensitivity analysis

We will perform sensitivity analysis to check robustness of pooled outcome results by removing qualified RCTs with low quality.

### 2.11 Publication bias

If the results of meta-analysis include more than 10 RCTs, we will conduct funnel plot to investigate the risk of publication bias,^[37]^ and Egger regression test to examine funnel plot asymmetry.^[38]^

### 2.12 Setting

There are no restrictions regarding setting of the study.

### 2.13 Dissemination and ethics

Findings of this study will be disseminated through a peer-reviewed journal and conferences. This study will not analyze individual patient data, thus no ethic approval is needed.

## 3 Discussion

ROP remains a leading cause of blindness in children worldwide.^[1,2]^ Despite a variety of treatments are available to manage this condition, its pathogenesis is still not completely elaborated. Studies suggested that ROP is associated with overproduction of VEGF.^[5,6]^ Conventional LT is reported to treat ROP because it can suppress VEGF overproduction.

Previous clinical trials have reported that LT can be utilized to manage ROP.^[14–28]^ However, no systematic review and meta-analysis has been conducted to assess this topic. Thus, it is very necessary to systematically and comprehensively explore the efficacy and safety of LT for ROP. The results of this study will provide up-to-date evidence to judge whether LT is effective or not for treating ROP. Its findings may provide evidence for clinician and further research.

## Author contributions

**Conceptualization:** Fei Wang, Li-xia Hao.

**Data curation:** Fei Wang, Li-xia Hao.

**Formal analysis:** Fei Wang, Li-xia Hao.

**Investigation:** Li-xia Hao.

**Methodology:** Fei Wang.

**Project administration:** Li-xia Hao.

**Resources:** Fei Wang.

**Software:** Fei Wang.

**Supervision:** Li-xia Hao.

**Validation:** Fei Wang, Li-xia Hao.

**Visualization:** Fei Wang, Li-xia Hao.

**Writing – original draft:** Fei Wang, Li-xia Hao.

**Writing – review & editing:** Fei Wang, Li-xia Hao.
